# Supplementary material for: Transcriptional inhibition after irradiation occurs preferentially at highly expressed genes in a manner dependent on cell cycle progression
Source: bioRxiv. 2024 Jun 11:2023.11.20.567799. Originally published 2023 Nov 20. Preprint. [Version 2] doi: 10.1101/2023.11.20.567799 (PMC10690177; doi:10.1101/2023.11.20.567799)

bioRxiv preprint doi: https://doi.org/10.1101/2023.11.20.567799; this version posted June 11, 2024. The copyright holder for this preprint (which was not certified by peer review) is the author/funder, who has granted bioRxiv a license to display the preprint in perpetuity. It is made available under aCC-BY 4.0 International license.

Figure 1-figure supplement 1. Transcriptional inhibition after irradiation and transcriptional restart after DNA repair in U2OS cells. A. U2OS cells were either incubated with EU or not, as indicated, and were irradiated (10 Gy) or not as indicated, followed by detection of the EU by click chemistry of a fluorophore, and immunofluorescence staining of gamma H2AX in the same cells and the DNA was detected by Hoechst staining.

Figure 1-figure supplement 2. Confirmation of knockdown of HIRA (A) and H3.3 (B). The samples were from the same experiments shown in Figure 1.

Figure 2-figure supplement 1. The mean intensities of the EU peaks shown in Figure 2D are indicated. The mean value of each sample was calculated by Flowjo software.

bioRxiv preprint doi: https://doi.org/10.1101/2023.11.20.567799; this version posted June 11, 2024. The copyright holder for this preprint (which was not certified by peer review) is the author/funder, who has granted bioRxiv a license to display the preprint in perpetuity. It is made available under aCC-BY 4.0 International license.

Figure 3-figure supplement 1. Nascent transcript levels before and after 30 minutes of IR. The samples from two independent experiments were used for the EU-seq and analyses in Fig. 3 and 4.

Figure 3-figure supplement 2. Screen shot from the UCSC browser of nascent transcripts. A. Nascent transcripts before and after IR over ribosomal DNA (rDNA). **B.** Nascent histone transcripts before and after IR over histone cluster 1.

Figure 3-figure supplement 3. Validation of nascent transcript levels of DEGs from EU-RNA seq by real-time quantitative RT-PCR. Samples from three independent experiments were used for the analyses. Ct value is presented to show the absolute amounts of EU labeled RNA transcripts from the same number of cells before and after 30 minutes of IR (10 gray). Significant difference after IR compared to before IR are indicated by asterisks, where ** indicates $p < 0.01$, * indicates $p < 0.05$ and ns indicates non-significant by students T-test.

Figure 5-figure supplement 1. CRISPR-Cas9 screen identifies genes promoting transcriptional inhibition after IR. A volcano plot of guide RNA changes between Eu high cells and unsorted cells. Labeled genes are some of those that have P adjust <= 10^{-6}.

Figure 5-figure supplement 2. Confirmation of knockdown of Nae1. gEV is an empty vector. The samples were from the same experiments shown in Figure 5D.

bioRxiv preprint doi: https://doi.org/10.1101/2023.11.20.567799; this version posted June 11, 2024. The copyright holder for this preprint (which was not certified by peer review) is the author/funder, who has granted bioRxiv a license to display the preprint in perpetuity. It is made available under aCC-BY 4.0 International license.

Figure 6-figure supplement 1. Analysis of CUL4A and CUL4B depletion. A. The western blot shows the CUL4A and CUL4B levels from the experiment shown in Fig. 6B. Additionally gRNAs were used to deplete CUL4A in cul4b cells and CUL4B in cul4a cells and their western blot analyses are also shown. **B.** The EU analysis of these double depleted cells is shown**. C.** Cell cycle analysis of the experiment shown in A and B and Fig. 6B.

Figure 6-figure supplement 2. Cell cycle analysis of cells treated with MLN4924 and RO3306. MLN4924 or RO3306 treatment leads cell cycle arrest in G_2 phase.

Figure 7-figure supplement 1. Quantification of cells with TOPBP1 expression before and after IR (10 Gy) in cells treated with MLN4924 and RO-3306. The samples were from the same experiments shown in Fig. 7F, 7G.

Supplementary File 1. Nascent RNA profiles of each gene using EU RNA-seq.

Supplementary File 2. Significantly enriched GO terms for up-regulated gene after irradiation.

Supplementary File 3. Significantly enriched GO terms for down-regulated genes after irradiation.

Supplementary File 4. Whole genome CRISPR-Cas9 screen detects the abundance of all gRNAs and target genes for EU high cells and unsorted cells.

bioRxiv preprint doi: https://doi.org/10.1101/2023.11.20.567799; this version posted June 11, 2024. The copyright holder for this preprint (which was not certified by peer review) is the author/funder, who has granted bioRxiv a license to display the preprint in perpetuity. It is made available under aCC-BY 4.0 International license.

Figure 1-figure supplement 2 Source Data 1. Original file for the Western blot analysis in Figure 1-Figure Supplement 1A (anti-HIRA and anti-GAPDH).

Figure 1-figure supplement 2 Source Data 2. PDF containing Figure 1-Figure Supplement 1A and original scans of the relevant Western blot analysis (anti-CUL4A, anti-CUL4B and anti-GAPDH) with highlighted bands and sample labels.

Figure 1-figure supplement 2 Source Data 3. Original file for the Western blot analysis in Figure 1-Figure Supplement 1B (anti-H3.3 and anti-GAPDH).

Figure 1-figure supplement 2 Source Data 4. PDF containing Figure 1-Figure Supplement 1B and original scans of the relevant Western blot analysis (anti-H3.3 and anti-GAPDH) with highlighted bands and sample labels.

Figure 5-figure supplement 2 Source Data 1. Original file for the Western blot analysis in Figure 5D (anti-Nae1 and anti-GAPDH).

Figure 5-figure supplement 2 Source Data 2. PDF of Western blot analysis in Figure 5D and original scans of the relevant Western blot analysis (anti-Nae1 and anti-GAPDH) with highlighted bands and sample labels.

bioRxiv preprint doi: https://doi.org/10.1101/2023.11.20.567799; this version posted June 11, 2024. The copyright holder for this preprint (which was not certified by peer review) is the author/funder, who has granted bioRxiv a license to display the preprint in perpetuity. It is made available under aCC-BY 4.0 International license.

Figure 6 Source Data 1. Original file for the Western blot analysis in Figure 6A (anti-CUL4A, anti-CUL4B and anti-GAPDH).

Figure 6 Source Data 2. PDF containing Figure 6A and original scans of the relevant Western blot analysis (anti-CUL4A, anti-CUL4B and anti-GAPDH) with highlighted bands and sample labels.

Figure 6 Source Data 3. Original file for the Western blot analysis in Figure 6C (anti-CUL4B, and anti-GAPDH).

Figure 6 Source Data 4. PDF containing Figure 6C and original scans of the relevant Western blot analysis (anti-CUL4B and anti-GAPDH) with highlighted bands and sample labels.

Figure 6-figure supplement 1 Source Data 1. Original file for the Western blot analysis in Figure 6-figure supplement 1A (anti-CUL4A, anti-CUL4B and anti-GAPDH).

Figure 6-figure supplement 1 Source Data 2. PDF containing Figure 6-figure supplement 1A and original scans of the relevant Western blot analysis (anti-CUL4A, anti-CUL4B and anti-GAPDH) with highlighted bands and sample labels.

Figure 1-figure supplement 1

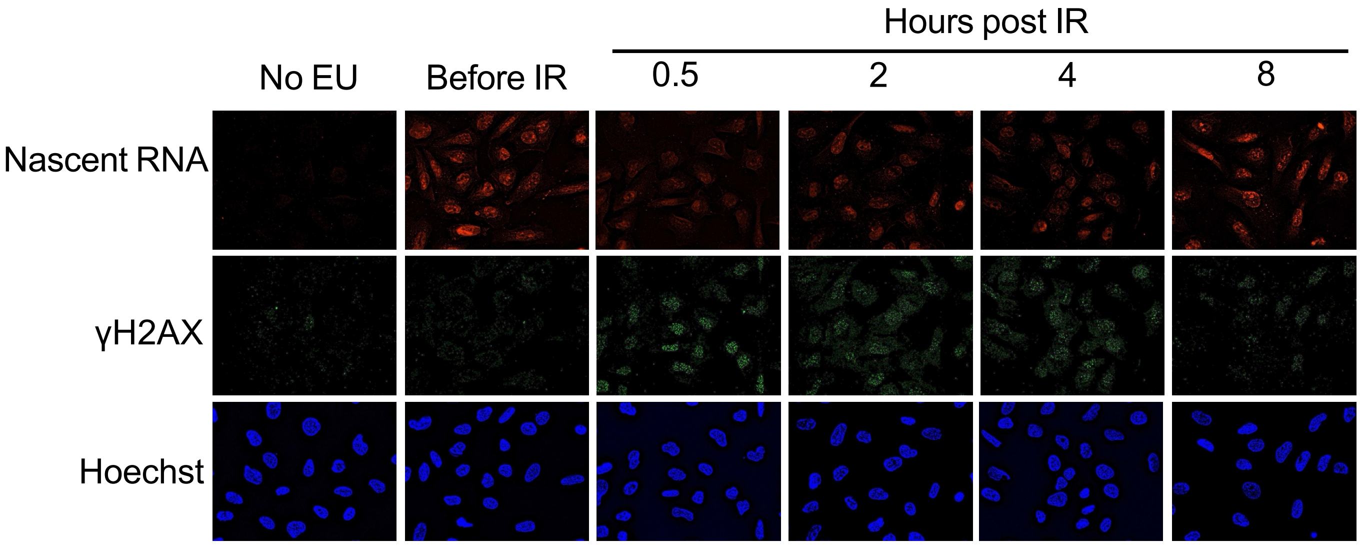

Figure 1-figure supplement 2

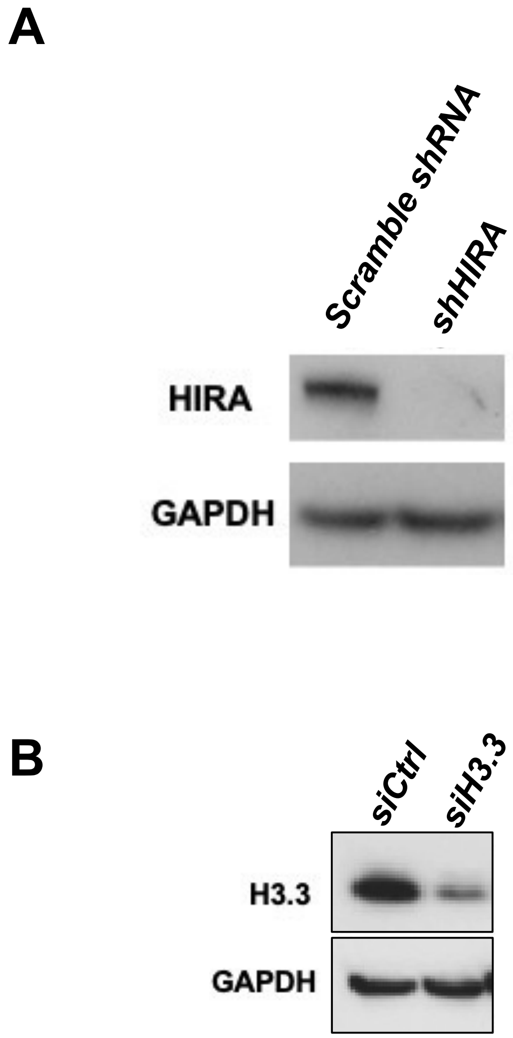

Figure 2-figure supplement 1

Dose	No EU	No IR	15 m	30 m	1 h	2 h	4 h
2 Gy	314	2690	1440	1261	1282	1640	2349
5 Gy	314	2690	1379	1169	1101	1345	2004
10 Gy	314	2690	1224	1094	1053	1347	1803

Figure 3-figure supplement 1

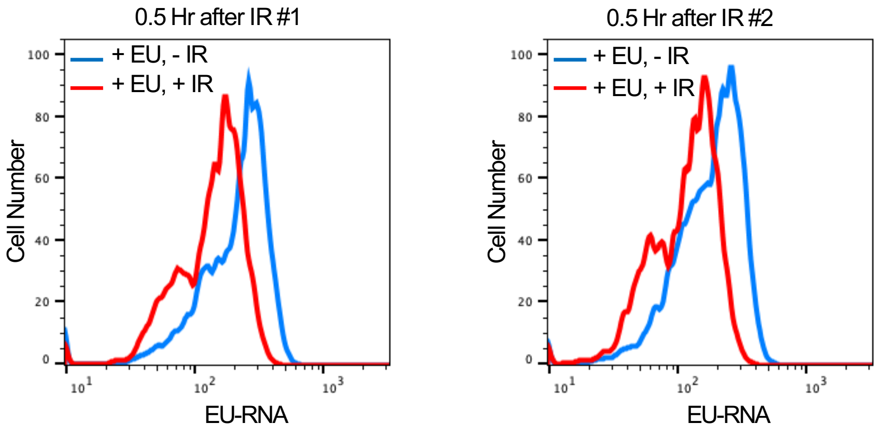

Figure 3-figure supplement 2

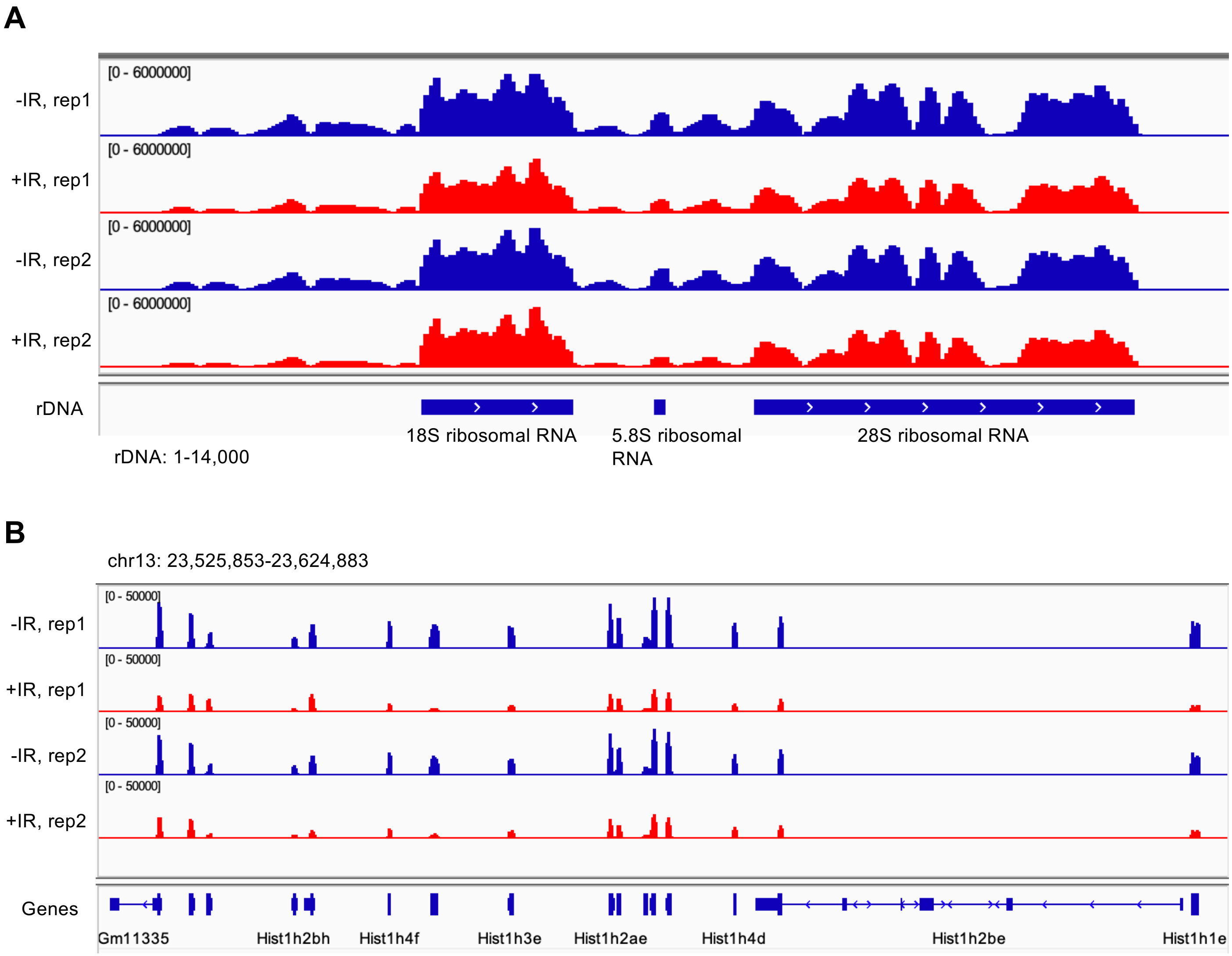

Figure 3-figure supplement 3

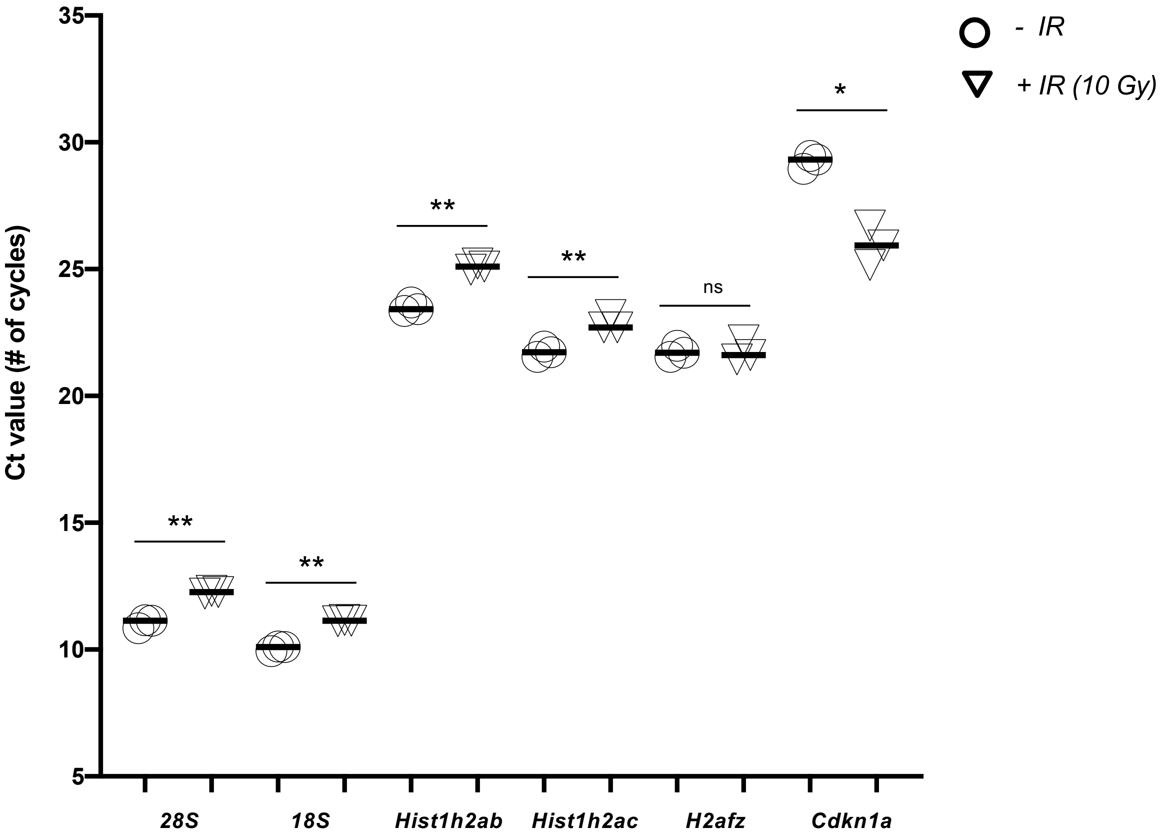

Figure 5-figure supplement 1

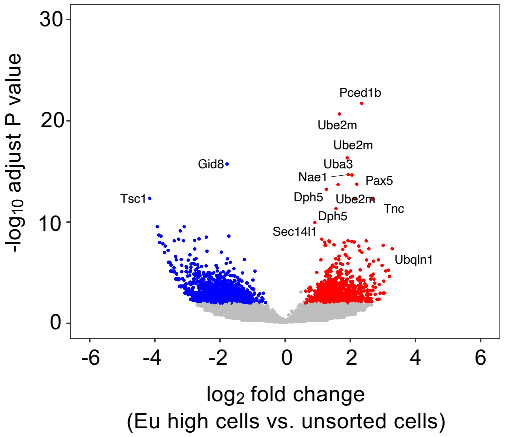

Figure 5-figure supplement 2

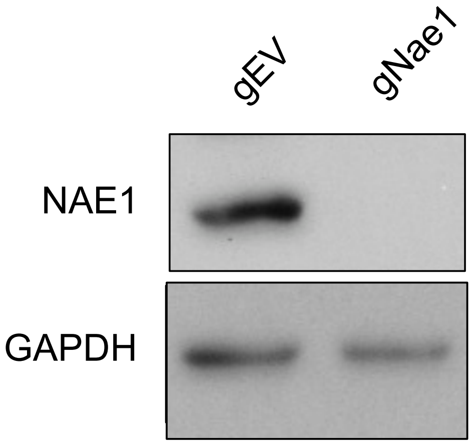

Figure 6-figure supplement 1

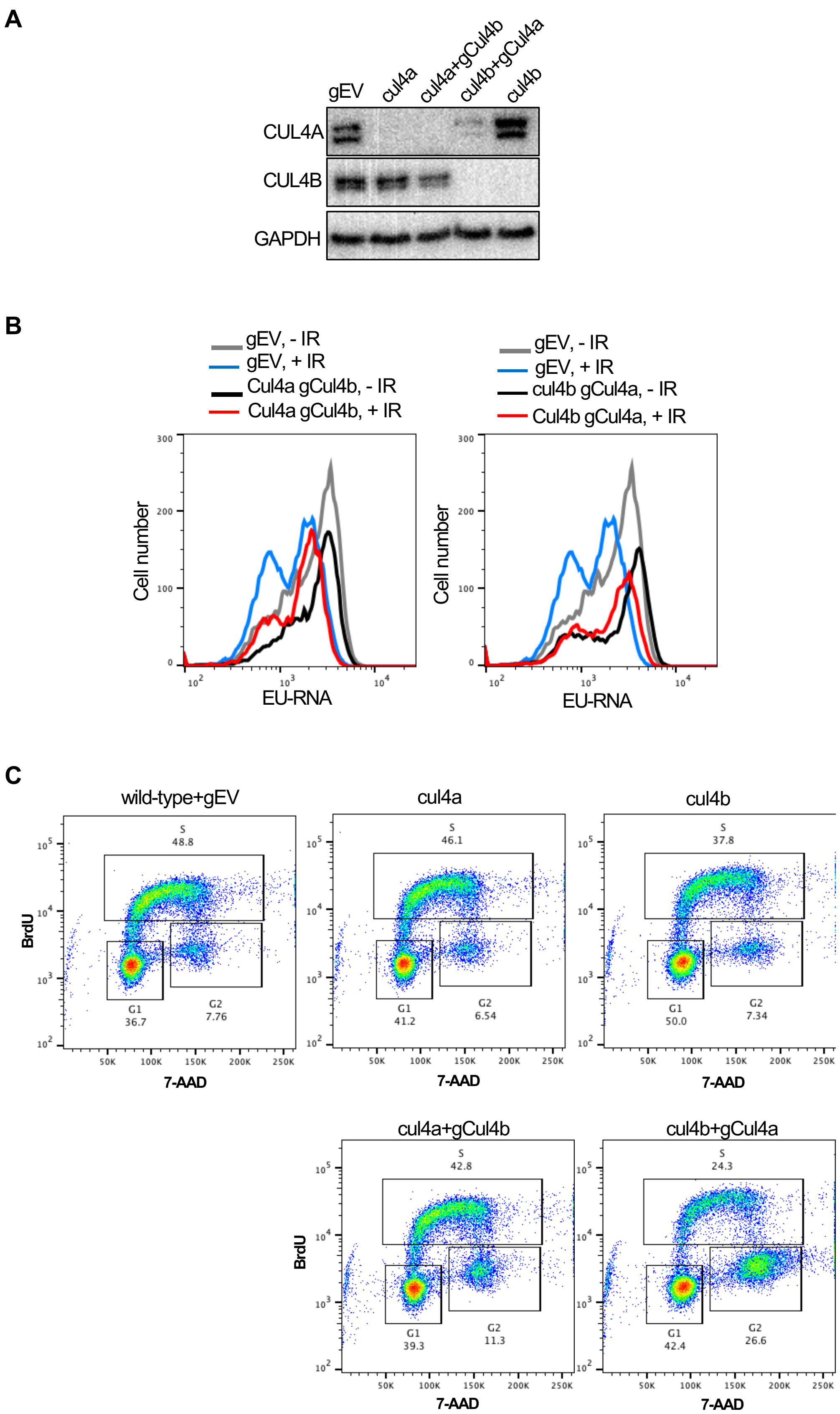

Figure 6-figure supplement 2

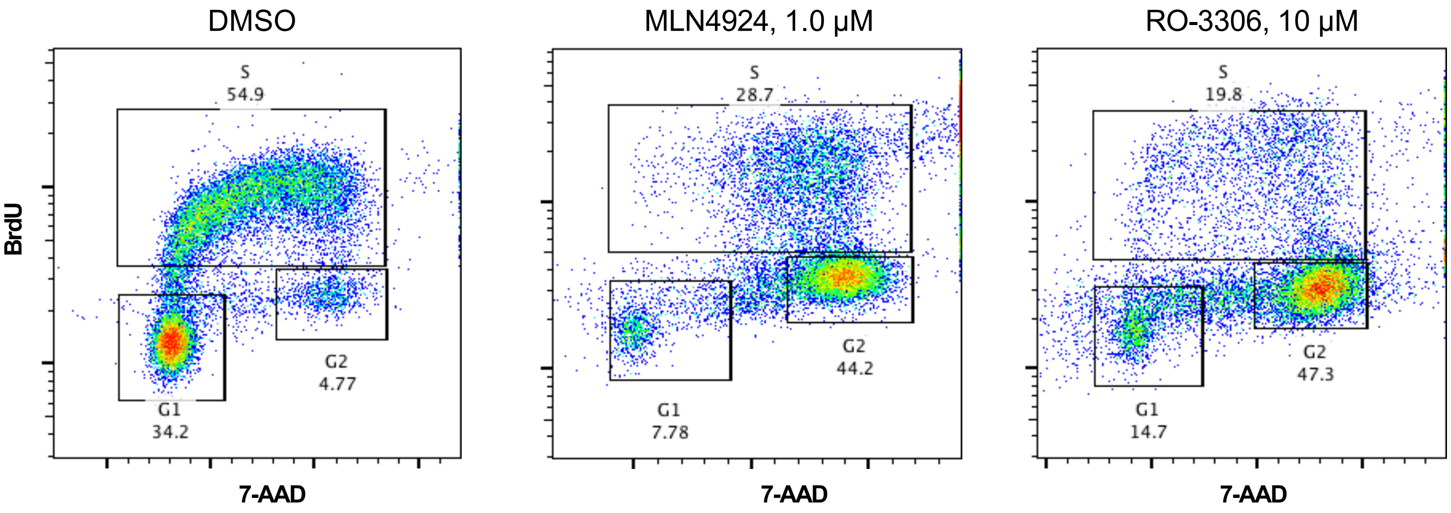

Figure 7-figure supplement 1

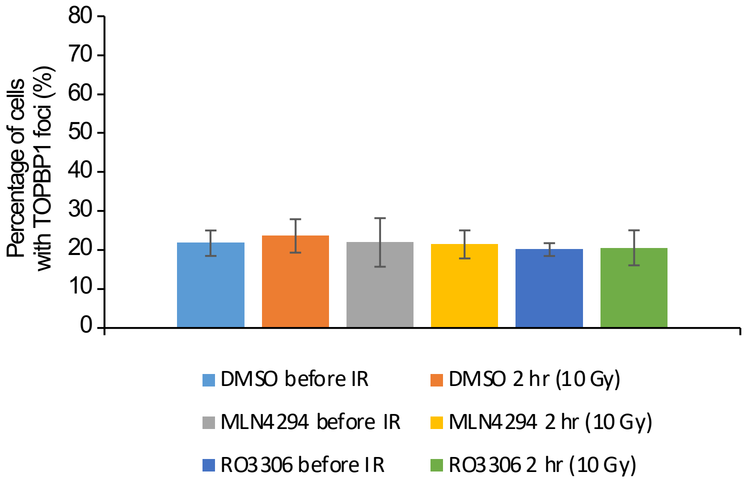

Supplement: 1 [file NIHPP2023.11.20.567799V2-supplement-1.pdf]
