# Supplementary material for: Transcriptional inhibition after irradiation occurs preferentially at highly expressed genes in a manner dependent on cell cycle progression
Source: bioRxiv. 2023 Nov 20:2023.11.20.567799. Preprint. [Version 1] doi: 10.1101/2023.11.20.567799 (PMC10690177; doi:10.1101/2023.11.20.567799)

Supp Fig. 1

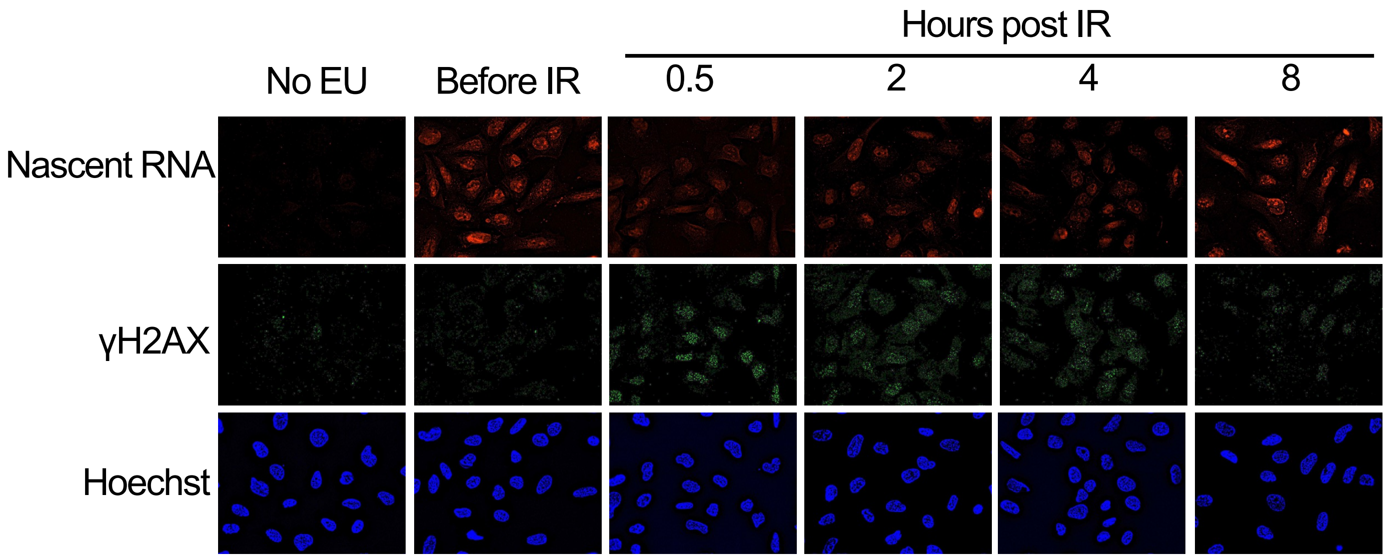

Hours post IR
No EU
Before IR
0.5
2
4
8
Nascent RNA
γH2AX
Hoechst

Supp Fig. 2

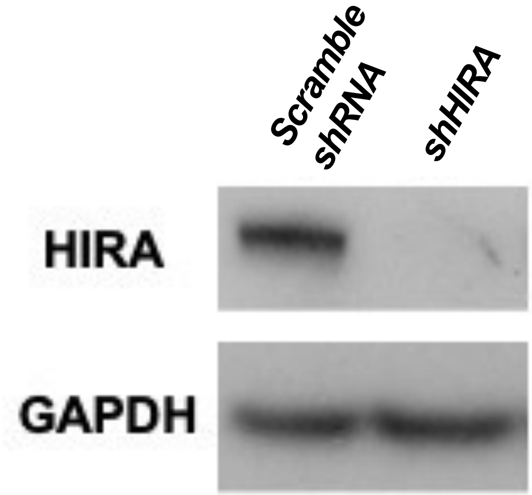

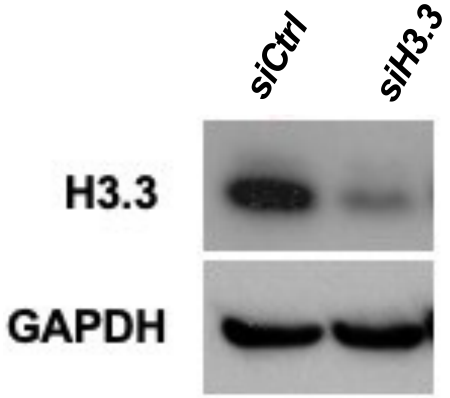

Supp Fig. 3

Dose	No EU	No IR	15 m	30 m	1 h	2 h	4 h
2 Gy	314	2690	1440	1261	1282	1640	2349
5 Gy	314	2690	1379	1169	1101	1345	2004
10 Gy	314	2690	1224	1094	1053	1347	1803

Supp Fig. 4

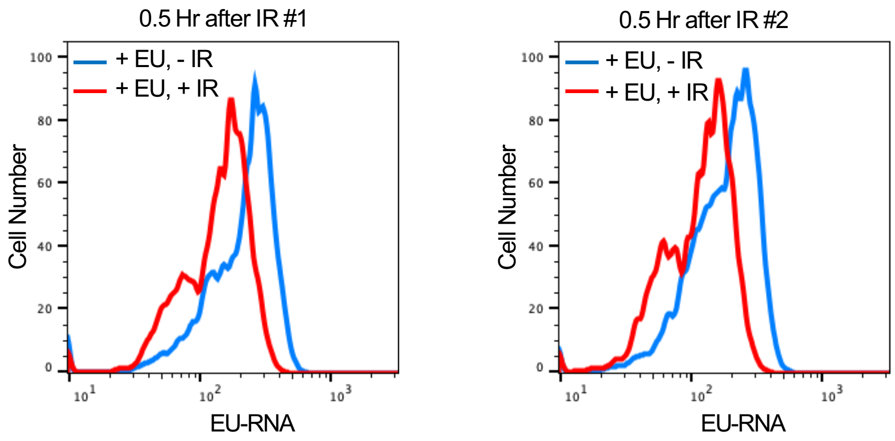

0.5 Hr after IR #1
0.5 Hr after IR #2
+ EU, - IR
+ EU, + IR
Cell Number
EU-RNA
0
20
40
60
80
100
10^1
10^2
10^3

Supp Fig. 5

A

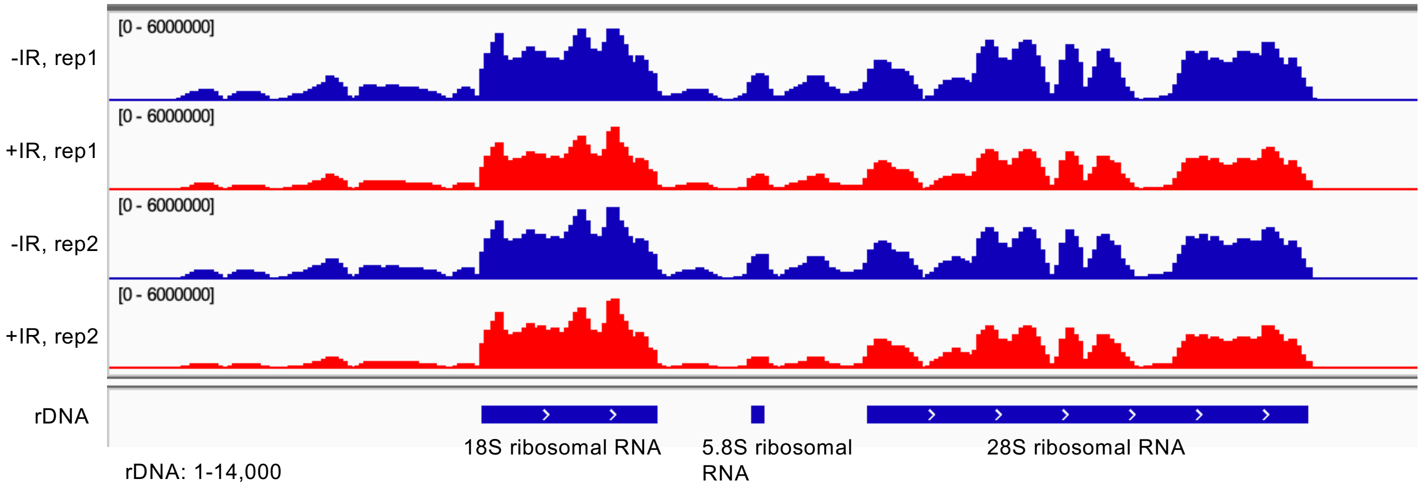
[0 - 6000000]
-IR, rep1
+IR, rep1
-IR, rep2
+IR, rep2
rDNA
18S ribosomal RNA
5.8S ribosomal RNA
28S ribosomal RNA
rDNA: 1-14,000

B

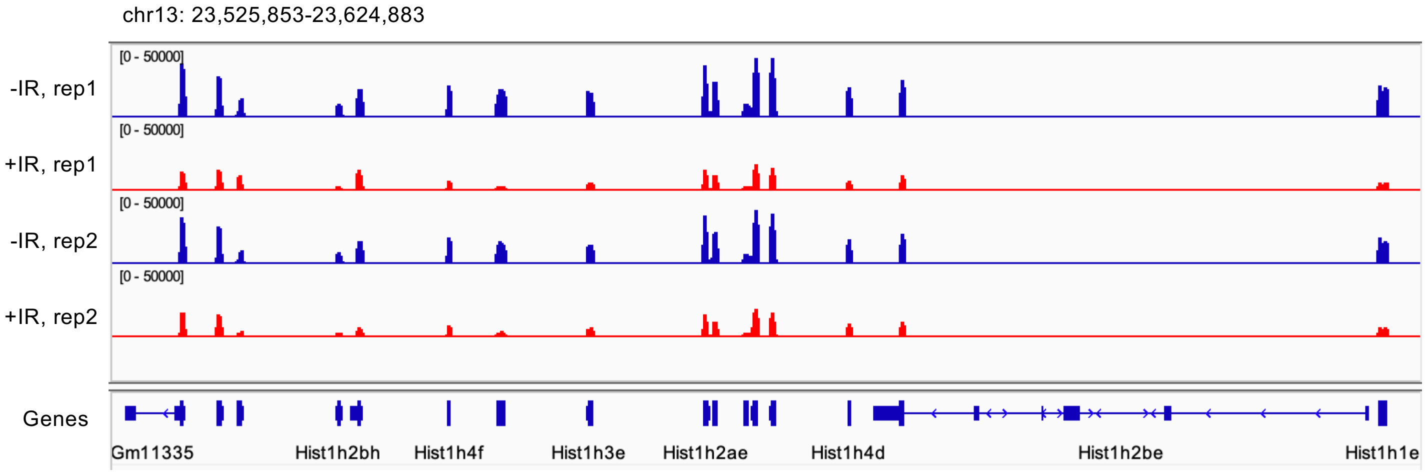
chr13: 23,525,853-23,624,883
[0 - 50000]
-IR, rep1
+IR, rep1
-IR, rep2
+IR, rep2
Genes
Gm11335
Hist1h2bh
Hist1h4f
Hist1h3e
Hist1h2ae
Hist1h4d
Hist1h2be
Hist1h1e

Supp Fig. 6

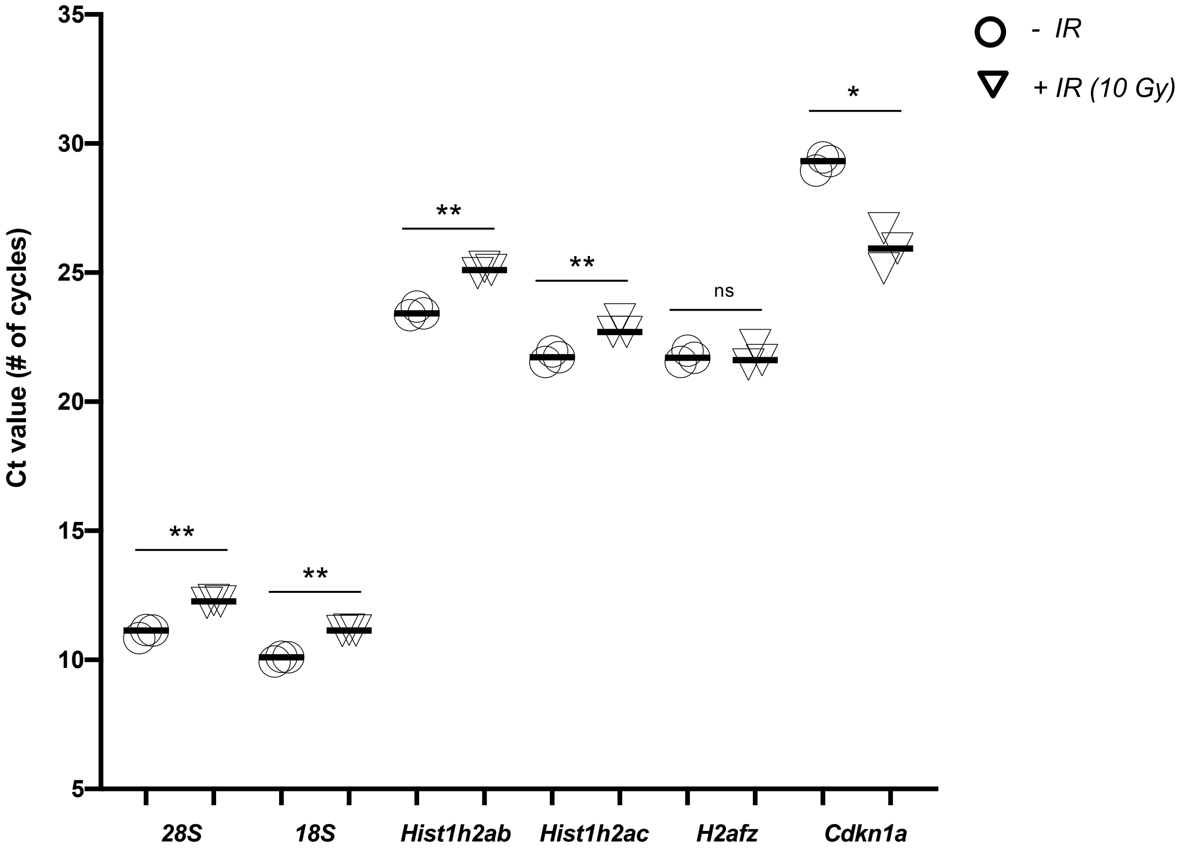

Ct value (# of cycles)
35
30
25
20
15
10
5
28S
18S
Hist1h2ab
Hist1h2ac
H2afz
Cdkn1a
**
**
**
**
ns
*
- IR
+ IR (10 Gy)

Supp Fig. 7

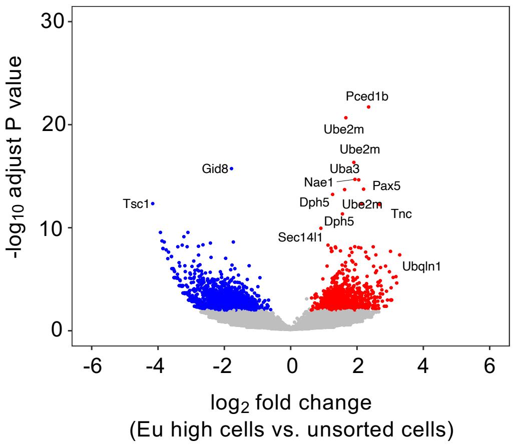
30
20
10
0
-log10 adjust P value
Pced1b
Ube2m
Ube2m
Uba3
Gid8
Nae1
Pax5
Tsc1
Dph5
Ube2m
Tnc
Dph5
Sec14l1
Ubqln1
-6
-4
-2
0
2
4
6
log2 fold change
(Eu high cells vs. unsorted cells)

Supp Fig. 8

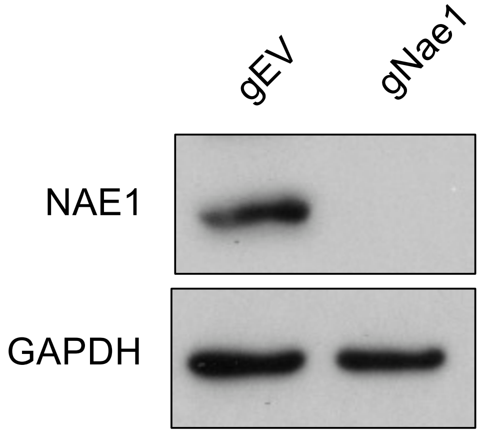

Supp Fig. 9

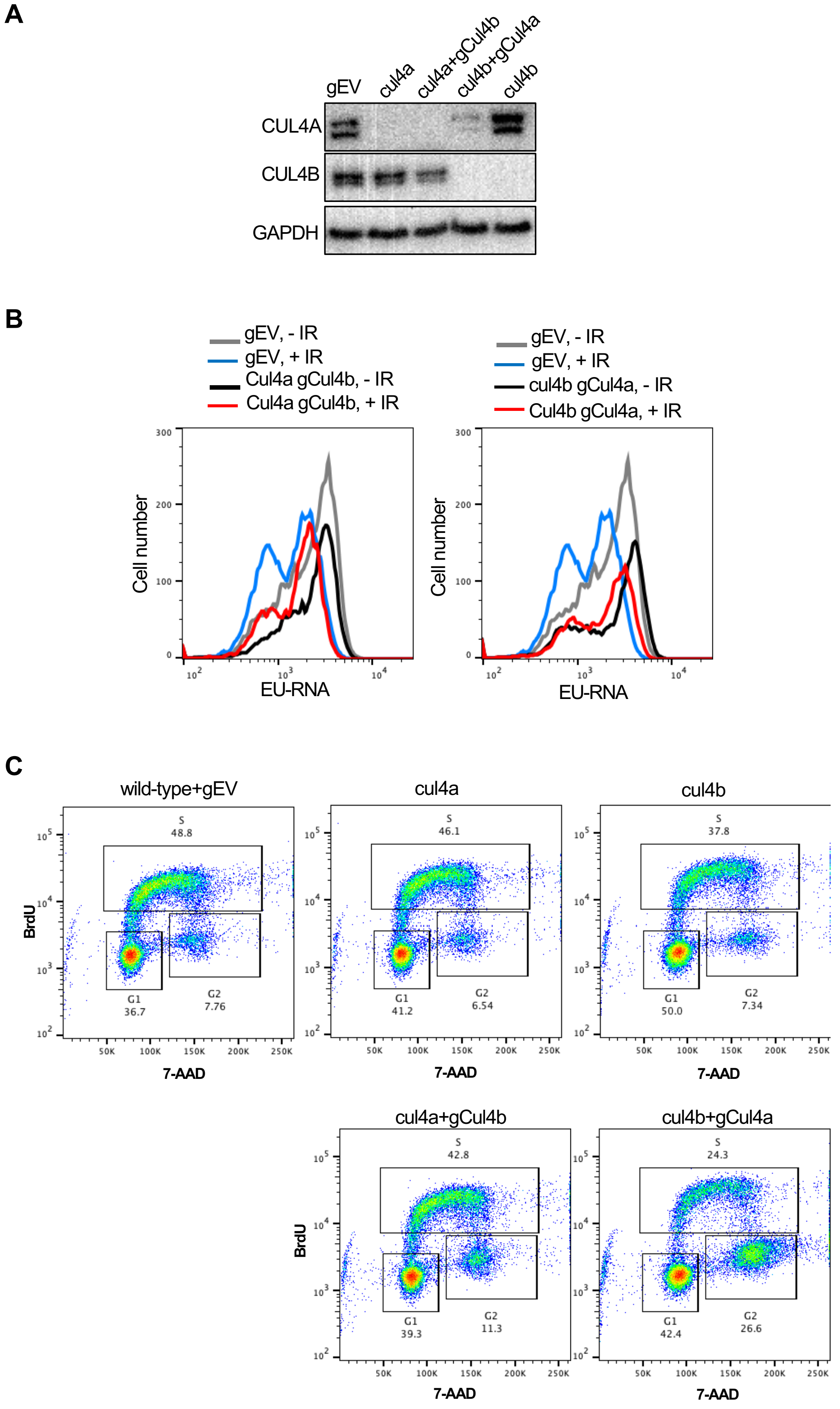
A
gEV
cul4a
cul4a+gCul4b
cul4b+gCul4a
cul4b
CUL4A
CUL4B
GAPDH
B
gEV, - IR
gEV, + IR
Cul4a gCul4b, - IR
Cul4a gCul4b, + IR
cul4b gCul4a, - IR
Cul4b gCul4a, + IR
Cell number
EU-RNA
C
wild-type+gEV
cul4a
cul4b
cul4a+gCul4b
cul4b+gCul4a
S
48.8
46.1
37.8
42.8
24.3
G1
36.7
41.2
50.0
39.3
42.4
G2
7.76
6.54
7.34
11.3
26.6
BrdU
7-AAD

Supp Fig. 10

A

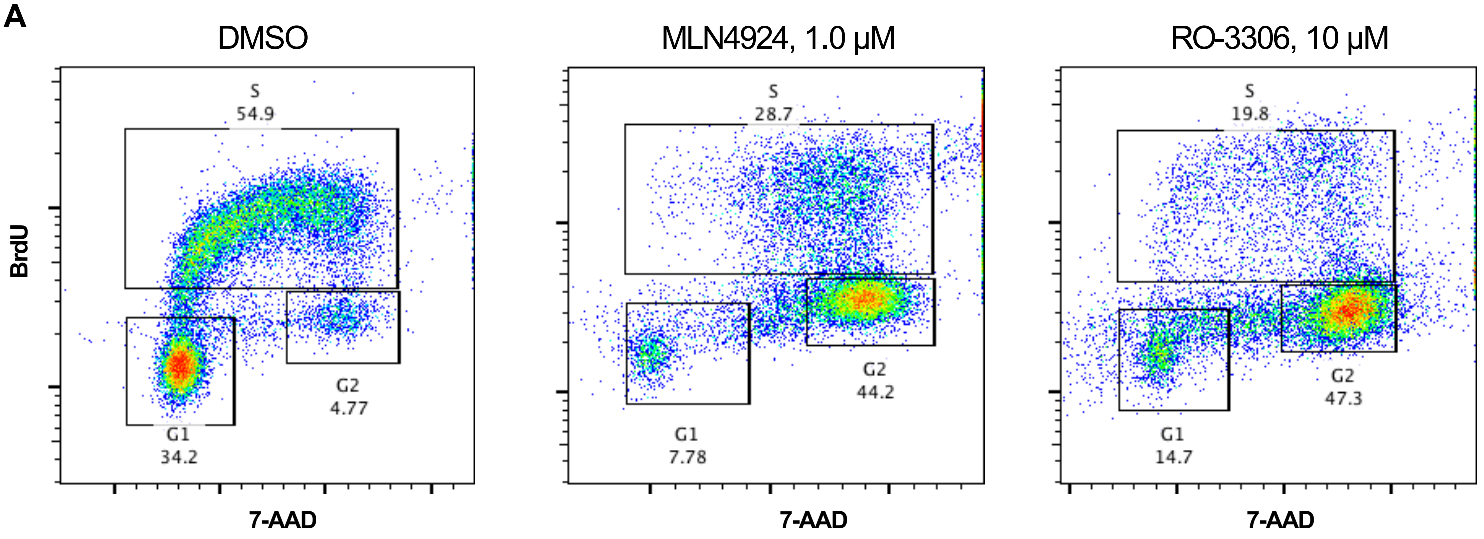

B

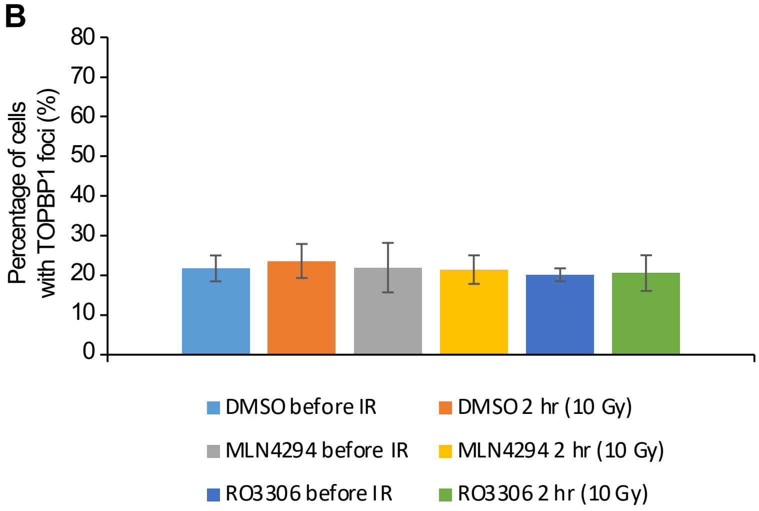

Supplement: Supplementary file 1 [file NIHPP2023.11.20.567799V1-supplement-1.pdf]
